# Revealing European cave shrimp diversity: a new species of *Spelaeocaris* (Decapoda, Atyidae) named through public participation

**DOI:** 10.3897/zookeys.1267.176622

**Published:** 2026-01-22

**Authors:** Jure Jugovic, Cene Fišer, Maja Zagmajster, Teo Delić, Valerija Zakšek

**Affiliations:** 1 University of Primorska, Faculty of Mathematics, Natural Sciences and Information Technologies, Glagoljaška 8, 6000 Koper, Slovenia University of Ljubljana Ljubljana Slovenia; 2 University of Ljubljana, Biotechnical Faculty, Department of Biology, SubBio Lab, Jamnikarjeva 101, 1000 Ljubljana, Slovenia University of Primorska Koper Slovenia

**Keywords:** Cave fauna, conservation, Dinaric Karst, endemism, public naming, subterranean

## Abstract

The family Atyidae, the world’s largest family of freshwater shrimps, is represented in Europe almost exclusively by subterranean species. The Dinaric Karst of the northwestern Balkans is among the most species-rich regions for atyids, hosting two genera, *Troglocaris* and *Spelaeocaris*. Recent speleobiological surveys in the southern Dinaric Karst of Bosnia and Herzegovina revealed a new cave shrimp species, described here as *Spelaeocaris electa* Jugovic & Zakšek, **sp. nov**. based on specimens collected during several field campaigns during 2013 and 2021. The epithet “electa” was chosen by a public vote, with the aim of involving local communities in the species naming process and raising awareness of subterranean biodiversity. Phylogenetic analyses, including species of the three European subterranean atyid genera (*Spelaeocaris*, *Troglocaris* and *Xiphocaridinella*) revealed its close relationship with *S. hercegovinensis*. Morphologically, *S. electa* Jugovic & Zakšek, **sp. nov**. is characterized by a weakly bilobed distal end of the telson, which is slightly concave in the centre, by a long rostrum, and the interchange of long and short spines on the appendix masculina. The discovery of *S. electa* Jugovic & Zakšek, **sp. nov**. highlights the exceptional subterranean biodiversity and high endemism of the Dinaric Karst, and emphasizes the need for continued taxonomic research and strengthened conservation efforts in this area.

## Introduction

We are currently undergoing the sixth mass extinction in Earth’s history, characterised by a dramatic loss of biodiversity driven by climate change and human activities (Barnosky et al. 2011) but see Wiens and Saban (2025). This biodiversity crisis is exacerbated by a parallel crisis of taxonomy, i.e. insufficient pace at which new species are discovered, described and recorded (de Carvalho et al. 2005; Engel et al. 2021). This challenge is even more pronounced in remote and inaccessible habitats, such as the subterranean ones, i.e. caves, interstitial or shallow subterranean habitats (Ficetola et al. 2019). Even in well-studied regions, incomplete inventories of subterranean habitats are more the rule than the exception, and the Dinaric Karst in the Balkan Peninsula, a global hotspot of groundwater biodiversity (Zagmajster et al. 2014), is one of them. Although the Dinaric Karst is one of the most intensively studied regions for subterranean fauna, having one of the longest research traditions in subterranean realm worldwide, the knowledge of its groundwater organisms remains scattered, and new species continue to be discovered regularly (e.g. Borko et al. 2022; Delić et al. 2025).

The Dinaric Karst is renowned for its extraordinary or remarkable freshwater subterranean taxa, such as the olm *Proteus anguinus* (see Recknagel et al. 2024), or filter-feeding animals such as the bivalve species complex *Congeria* (see Bilandžija et al. 2013), the tube worm *Marifugia cavatica* (see Kupriyanova et al. 2009) or the cnidarian *Velkovrhia enigmatica* (see Zagmajster et al. 2013). Among invertebrates, cave shrimps stand out for their size and elegance, and they are relatively common and easy to observe in caves along the Dinaric Karst.

The Atyidae is the largest family of freshwater shrimps, with members inhabiting a wide range of habitats, from surface to subterranean (von Rintelen et al. 2012) and from freshwater to mixohialine (De Grave et al. 2008; Fransen and De Grave 2011). In Europe, the atyids live almost exclusively in subterranean waters. Two monotypic genera – *Gallocaris* (Sket and Zakšek 2009) in France and *Ficticaris* in Serbia (Jugovic et al. 2019) – occupy isolated areas, whereas the highest species diversity of cave shrimps is found in the Caucasus with the genus *Xiphocaridinella* (Marin and Turbanov 2021) and in the Dinaric Karst with the genera *Troglocaris* and *Spelaeocaris* (see Zakšek et al. 2007, 2009; Marin and Turbanov 2021). In the last decade, extensive explorations in the Caucasus have led to the description of numerous new *Xiphocaridinella* species (e.g. Marin 2017; Marin and Sinelnikov 2017; Marin and Turbanov 2021), and many new species and subspecies have also recently been described from the Dinaric Karst (Sket and Zakšek 2009; Jugovic et al. 2012). However, Dinaric Karst still harbours species that have long remained overlooked, particularly in less-studied southern karstic areas.

Here, we focused on cave shrimps from the southern Dinaric Karst, specifically the genus *Spelaeocaris*. In contrast to the genus *Troglocaris*, whose diversity was extensively studied using molecular phylogenetic approaches (Zakšek et al. 2007, 2009), *Spelaeocaris* received much less research attention. We have thoroughly explored molecular diversity within the genus *Spelaeocaris*, compared it with the other two cave shrimp genera in Europe, *Troglocaris* and *Xiphocaridinella*, and assessed their phylogenetic relationships. Based on an integrative approach, combining morphological examination and molecular data (mitochondrial and nuclear DNA), we describe here a new species, *Spelaeocaris electa* sp. nov. We report on a participatory taxonomic approach that involved both local communities and speleobiologists in the species-naming process, thereby helping to raise public awareness of the region’s subterranean biodiversity.

## Materials and methods

### Sampling and distribution data

Specimens of the new species were collected with a hand net within 2013–2021 (18 October 2013, 25 April 2015, 23 October 2016, 14 March 2021, and 3 October 2021) in the caves of the southeastern Dinaric Karst (Bosnia and Herzegovina and Croatia; see Suppl. material 1 for details). To assess its phylogenetic position additional cave shrimp species were sampled. They were stored in 96% ethanol for both molecular and detailed morphological examination. Each individual used in molecular and/or morphological analyses was assigned a unique voucher number (Suppl. material 1). The specimens, including the holotype TB362 and paratype TB379, are stored in the zoological collection at the University of Ljubljana, Biotechnical Faculty, Department of Biology, Slovenia, paratype TB287 (CNHM 1833) and specimen TB263 (CNHM 1834) in the crustacean collection in the Croatian Natural History Museum (Zagreb), and paratype TB380 (INVA000152) and specimen TB377 (INVA000153) in the collection in the Zemaljski muzej Bosne i Hercegovine (Sarajevo).

Distributional data containing literature and unpublished data of all considered species in the Dinaric Karst were obtained from SubBioDB (https://db.subbio.net/), a distributional database on subterranean biology, managed by SubBioLab, University of Ljubljana.

### Morphological analysis

Specimens were dissected, and photographs of the body parts were taken under a stereomicroscope (Leica EZ4) equipped with a digital camera (Sony DXC390P) using the software LAZ EZ Ink. Measurements of morphological characters followed 73 metric and 17 meristic characters (for a list of characters, see Jugovic et al. 2010a, 2010b, 2011, 2012). Vector drawings were produced from microphotographs using a graphic tablet (Wacom, Cintiq 13HD Creative Pen Display) with the freeware Krita 4.1.1. Postorbital carapace length (CL) was used as a standardized measure of specimen size and as a reference measure for length relations (Jugovic et al. 2010b, 2011, 2012). The abbreviation RL is used for the rostrum length, measured from the tip of the rostrum to the postorbital margin. The rostral dentition formula:

(*X* + *Y*) / *Z*

denotes the number of dorsal rostral teeth (*X*), the number of dorsal teeth on the carapace (*Y*), and the number of ventral rostral teeth (*Z*). For reference, see Jugovic et al. (2010b: fig. 2), where *X*, *Y*, and *Z* correspond to ROT1, ROT2, and ROT3, respectively. In the description, values measured in the holotype are reported, with ranges (min–max) for male (♂♂) and female (♀♀) individuals, including subadults but excluding juveniles unless explicitly stated otherwise. The abbreviation SA is used for subadults.

We quantified sexual size dimorphism using the carapace length dimorphism index (*SDI*) of Lovich and Gibbons 1992, calculated as follows:

*SDI* = (CL_m_ / CL_f_) − 1

The *SDI* is zero if both sexes have the same body length, it is positive if males are longer than females, and negative if females are longer than males. To avoid bias due to small sample sizes and the inclusion of immature specimens (Saclier et al. 2024), we calculated *SDI* using the maximum body length of sexually mature specimens rather than the mean body length of all available specimens. Maximum body length estimates the size of fully-grown individuals and is often the only measurement given in species descriptions (Jugovic et al. 2024).

Sexual dimorphism in the lengths of appendages (antennae and pereiopods I–V) was tested using a One-way T-test. Statistical significance was set at p < 0.05. When significant differences were detected, results are reported as means ± standard error (SE).

### Molecular and phylogenetic analysis

In addition to 29 specimens with pre-existing sequences (Zakšek et al. 2007, 2009), 52 cave shrimp specimens from the Dinaric Karst and Caucasus were sequenced *de novo* for the purpose of this study (Suppl. material 1). Pleopods III, IV or V were used for molecular analysis and genomic DNA was isolated using the GeneElute Mammalian Genomic DNA Miniprep Kit (Sigma-Aldrich, USA). Two mitochondrial (cytochrome oxidase I – COI and 16S rDNA) and one nuclear gene partition (Internal Transcribed Spacer - ITS) were amplified in PCR reaction using primers and protocols described in Suppl. material 2. The PCR products were purified using Exonuclease I and FastAP (Thermo Scientific, USA) and sequenced in both directions with amplification primers by Macrogen Europe (Amsterdam, The Netherlands). Sequence chromatograms were visually checked, assembled and edited in Geneious v. 11.1.5 (Biomatters, New Zealand), aligned using MAFFT v. 7 (Katoh and Standley 2013), using the E-INS-I algorithm, and partitions were concatenated in Geneious. The total length of the concatenated dataset was 2223 bp. The most probable substitution model and partitioning scheme (see Suppl. material 2) were selected in Partitionfinder v. 2.1. (Lanfear et al. 2012).

In order to assess the phylogenetic position of the new *Spelaeocaris* species, we compiled a dataset of 79 specimens from the genera *Troglocaris*, *Spelaeocaris* and *Xiphocaridinella*. Two additional specimens of *Gallocaris inermis* and *Dugastella valentina* were used as outgroups. The list of taxa and specimens, the origin of the samples, and the GenBank Accession Numbers can be found in the Suppl. material 1. Phylogenetic relationships were reconstructed using Bayesian inference (BI) with partition-specific settings in MrBayes v. 3.2.7 (Ronquist and Huelsenbeck 2003) and maximum likelihood (ML) in IQ-tree (Nguyen et al. 2015). Bayesian MCMC tree search with two independent runs with four chains each was run for 10 million generations; trees were sampled every 1000^th^ generation. After reaching the stationary phase, the first 25% of trees were discarded, and a 50% majority rule consensus tree was calculated from the remaining trees. The BI analyses were run on the CIPRES Science Gateway (https://www.phylo.org/) and ML analyses on IQ-tree web server (Trifinopoulos et al. 2016). Genetic distances in the COI barcoding gene were estimated between *Spelaeocaris electa* sp. nov. and other *Spelaeocaris* species using the Kimura-2-parameter (K2P) model in MEGA11 (Tamura et al. 2021).

### Selecting the species name

Increasing public awareness is of the importance of subterranean biodiversity and its conservation in the southeastern Dinaric Karst was a central part of the SubBIOCODE project (led by SubBioLab, University of Ljubljana; and financed by CEPF – Critical Ecosystem Fund; 2019–2022). One of the public awareness activities was to actively involve a broad audience in the species’ name selection process. We proposed three potential names, each accompanied by a justification, and invited the public to express their preference. Voting was conducted both online and in person. An online voting was possible via Facebook 8–22 April 2022. Within this period, in-person voting was also possible, during the 3^rd^ Dinaric Symposium on Subterranean Biology in Trebinje, Bosnia and Herzegovina (9–10 April 2022). For the on-site voting, we distributed printed postcards presenting species with an attached ballot (Suppl. material 2: fig. S1).

## Results

### Phylogenetic analyses

**Figure 1.**
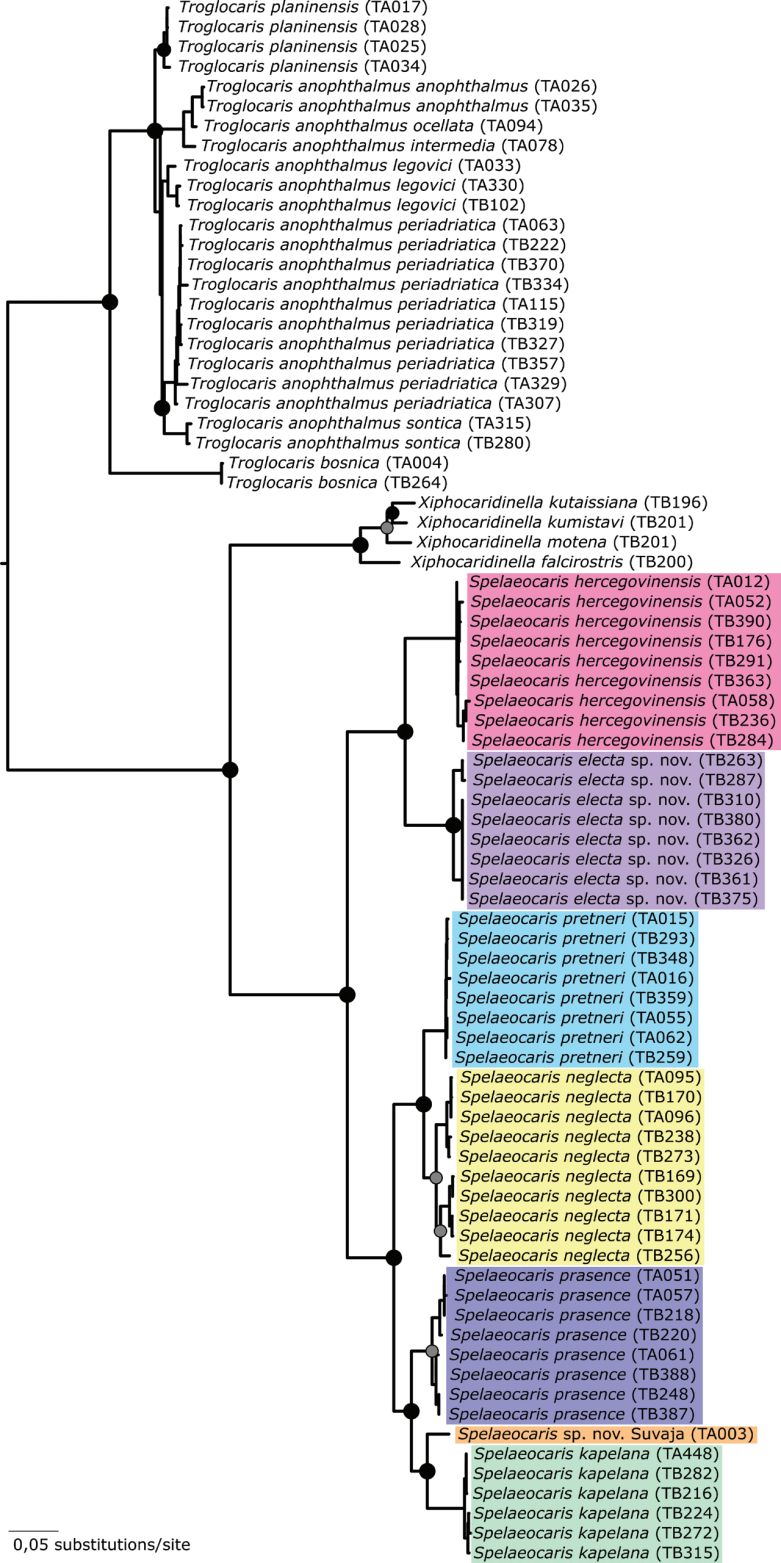
Phylogenetic relationships among the cave shrimps from the genera *Spelaeocaris*, *Xiphocaridinella* and *Troglocaris*. The tree was constructed using Bayesian inference on concatenated mitochondrial (COI and 16S rDNA) and nuclear (ITS2) dataset. The posterior probabilities for the nodes are shown as circles: black = 1 and grey > 0.95. The topology and a high main node support was also recovered with maximum likelihood tree (data not shown). The tree was rooted using *Gallocaris inermis* and *Dugastella valentina* (not shown).

Phylogenetic relationships among and within the genera *Troglocaris*, *Spelaeocaris* and *Xiphocaridinella*, inferred from a concatenated mitochondrial and nuclear data matrix, were fully resolved and concordant with the topologies derived from individual loci (not shown). All nominal *Spelaeocaris* and *Troglocaris* species from the Dinaric Karst, represented and sequenced by multiple individuals, formed monophyletic groups, mostly with high posterior probabilities and bootstrap support (Fig. 1). This also applies to *Spelaeocaris electa* sp. nov., which is a sister species to *S. hercegovinensis*.

The Kimura 2-parameter (K2P) genetic distances based on the mitochondrial COI barcoding region among species of *Spelaeocaris* ranged from 8.4% to 21.1% (Suppl. material 2: table S1). The K2P distance between *Spelaeocaris electa* sp. nov. and its sister species *S. hercegovinensis* is 15.7%, while the species pair *S. neglecta* and *S. pretneri* exhibits the lowest genetic distance (8.4%). There are some genetic differences also within *Spelaeocaris electa* sp. nov.: 2.07% between the specimens from the northern part of the area (Betina jama and Tomina jama; “jama” meaning a cave) and the southern part of its distribution range (see also Fig. 1). This level of intraspecific divergence is similar to those usually observed in some other crustaceans on species level (Allison et al. 2024) or even exceeds them in other barcoding studies (Matzen da Silva et al. 2011).

Samples are deposited in the zoological collection at the University of Ljubljana, Biotechnical Faculty, Department of Biology, Slovenia, in the zoological collection in the Croatian Natural History Museum (**CNHM**, Zagreb), and in the collection in Zemaljski muzej Bosne i Hercegovine (Sarajevo).

### Species description

#### Spelaeocaris electa

Taxon classificationAnimaliaDecapodaAtyidae

Jugovic & Zakšek sp. nov.

2E1C3D3B-227E-5B7B-AAA0-1C0D7952071C

https://zoobank.org/58392985-439F-4CE2-AA5C-AEA87E4DBDFC

Figs 1, 2, 3

##### Type material.

***Holotype***: • adult male, CL 6.6 mm (voucher number TB362), Buljovica (cave), Žakovo, Trebinje, Bosnia and Herzegovina (42.80251°N, 18.13804°E), 14 March 2021, coll. B. Rexepi, Š. Borko, E. Premate, A. Pekolj. ***Paratypes***: • 2 adult males and 1 subadult male – adult male CL 7.0 mm (TB379), Plitica (cave), Dračevo, Popovo polje, Bosnia and Herzegovina (42.85412°N, 18.06468°E), 3.10.2021 coll. T. Delić, B. Rexhepi, B. Jalžić, E. Premate, H. Recknagel; • adult male CL 6.4 mm (TB380), same data as for TB379; • subadult male CL 4.8 mm (TB287), Tomina jama, Croatia (43.02601°N, 17.58625°E), 23 October 2016, coll. B. Jalžić. ***Allotype***: • adult female CL 6.6 mm (TB361), collection data same as holotype.

##### Other material examined.

• 6 adult females, 2 subadult females, 2 juvenile females – adult female CL 6.6 mm (TB263), Betina velika jama, Kokorići, Croatia (43.19445°N, 17.32250°E), 18 October 2013 coll. T. Delić; • adult female CL 7.5 mm (TB377), adult female CL 7.2 mm (TB378), adult female CL 6.2 mm (TB386); adult female CL 7.0 mm (TB410), adult female CL 6.4 mm (TB383), collection data for all: Plitica (cave), Dračevo, Popovo polje, Bosnia and Herzegovina, 3 October 2021, coll. T. Delić, B. Rexhepi, B. Jalžić, E. Premate, H. Recknagel; • sub-adult female CL 6.1 mm (TB326); subadult female CL 5.1 mm (TB310), data for both: Baba pećina, Strujići, Bosnia and Herzegovina (42.90308°N, 18.00683°E), 25 April 2015, coll. T. Delić, D. Škufca, Š. Borko, A. Zamolo; • juvenile female CL 4.9 mm (TB411), Plitica (cave), Dračevo, Popovo polje, Bosnia and Herzegovina, 3 October 2021, coll. T. Delić, B. Rexhepi, B. Jalžić, E. Premate, H. Recknagel; • juvenile female CL 4.7 mm (TB375), Plitica (cave), Dračevo, Popovo polje, Bosnia and Herzegovina, 14 March 2021, coll. T. Delić, M. Zagmajster, A. Kos, H. Recknagel.

##### Diagnosis.

*Spelaeocaris electa* sp. nov. is a species of cave shrimps (depigmented and blind) of the family Atyidae. It is characterised by long rostrum (Fig. 5) that overreaches (> 1.1×) antennular length and bears numerous teeth (12–17+4–7/3–11), supra- and suborbital spines are present. Distal extension of antenna I segment is pointed, maxilliped-I exopodital lobe is narrow, and gradually narrowing into a distal flagellum; chelae and dactyli are narrow but with well-developed excavations on distal end of carpi (segments 5) of pereiopods I and II; only pereiopods III and IV (but not pereiopods V) are widened distally in mature males; male pleopods I and II protopodites bear numerous short spiniform setae along their inner margins, male pleopod I endopodite has oval and lobate lamina elongated into a finger-like appendix interna bearing a group of retinacular hooks, with a row of simple and plumose setae along inner and outer margins, respectively, without setation along more or less well developed distal lobe; male pleopod II appendix masculina is spindle-shaped, with numerous spiniform setae on its surface, of which the longest setae are longer than the width of appendix interna; appendix masculina is ~2× as long as half the thinner appendix interna; telson is weakly bilobed (Fig. 5), with specific arrangement of spiniform setae along its distal margin (from the margins towards the centre): one pair of shortest dorsolateral spinules, one pair of longest spines (at least 1.5× longer than any other spines), three pairs of intermediately long spines (each of them longer than previous in direction towards the centre), and a group of shortest two pairs (longer only than distolateral pair of spinules) positioned at the slightly concave centre of telson margin.

##### Description of male holotype (first reported value) and other material (values in parentheses, where available).

**Figure 2.**
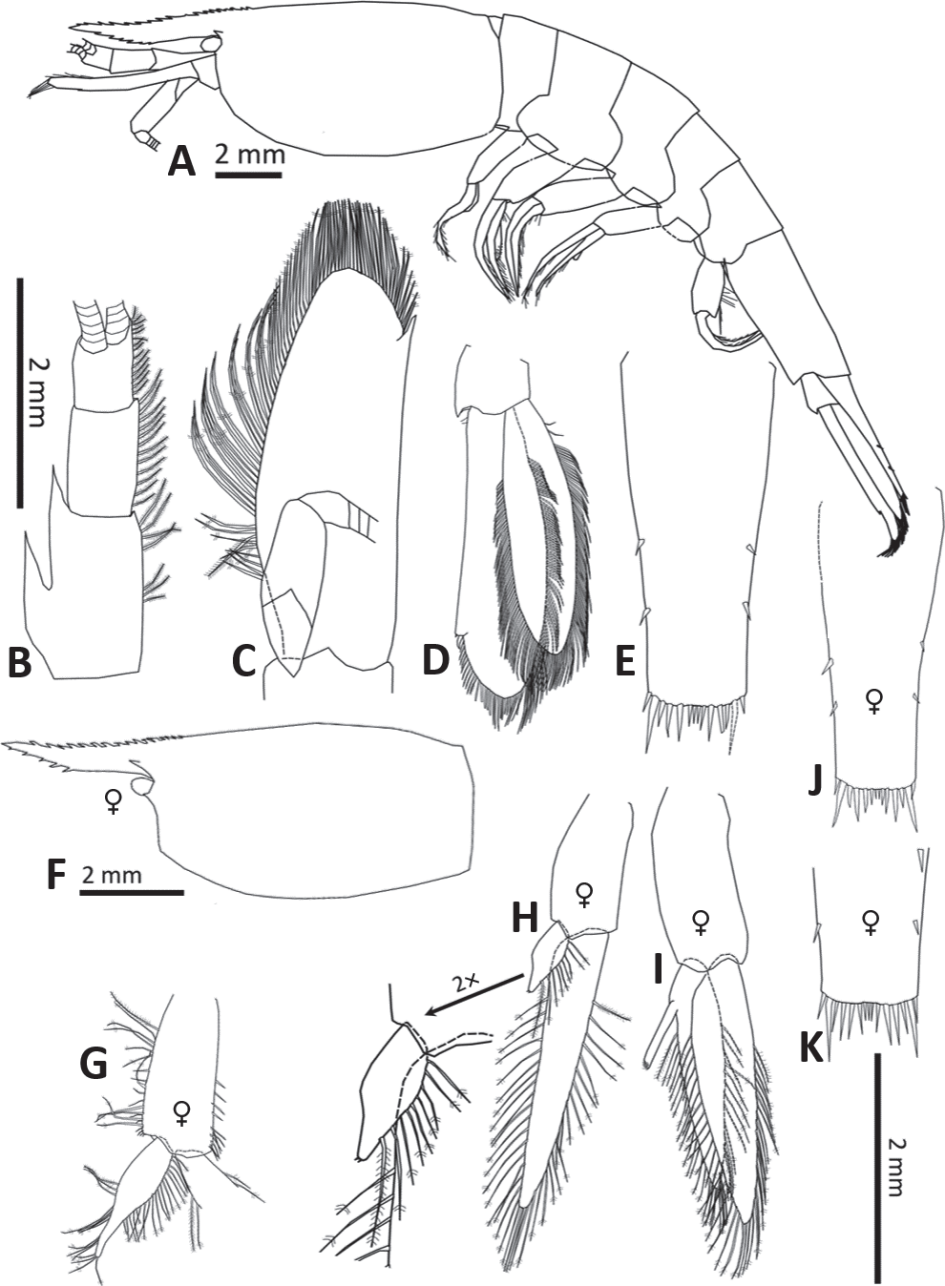
*Spelaeocaris electa* sp. nov. **A**. Body; **B**. Antenna I (antennula); **C**. Antenna II (antenna); **D**. Uropod (all holotype male, CL 6.6 mm, TB362, Buljovica (cave), Žakovo, Trebinje, Bosnia and Herzegovina); **E**. Telson, **F**. Carapace (female CL 6.6 mm, TB361, data same as holotype); **G**. Pleopod I (female CL 7.2 mm, TB378, Plitica (cave), Dračevo, Popovo polje, Bosnia and Herzegovina); **H**. Pleopod I (female CL 6.6 mm, TB361, data same as holotype); **I**. Pleopod II (female CL 6.6 mm, TB361, data same as holotype); **J**. Telson (female CL 6.6 mm, TB361, data same as holotype); **K**. Telson (female CL 7.5 mm, TB377, Plitica (cave), Dračevo, Popovo polje, Bosnia and Herzegovina); ♀ – female (not designated – male).

***Cephalothorax and cephalic appendages***. The carapace surface is smooth and depigmented. Its length in the holotype is 6.6 mm (♂♂ 4.8 mm (SA, *n* = 1), 6.5–7.0 mm, *n* = 3; ♀♀ 6.2–7.5 mm (*n* = 7), median 6.6 mm, SA females (*n* = 2) 5.1 mm and 6.1 mm, juvenile females 4.7–4.9 mm (*n* = 2)). The rostrum is long (Fig. 2A), in the holotype extending 1.2× beyond the antennular peduncle (♂♂ 1.17–1.22; ♀♀ 1.29–1.99); it is approximately as long as or slightly overreaching the scaphocerite (♂♂: 1.00–1.04; ♀♀: 1.04–1.61). The rostrum is 0.56× the length of the carapace in the holotype (♂♂: 0.56–0.61; ♀♀: 0.49–0.82). Rostral dentition in holotype follows the formula 12+6/7 (♂♂: TB379 has a partially broken rostrum with ≥ 10+5/ ≥ 4 teeth; TB380 has 12+4/9; TB287 has a completely broken rostrum but 4 dorsal teeth on the carapace behind the eyestalks; ♀♀: (11–17)+(4–7)/(5–11); the smallest juvenile female (TB375) shows only three ventral rostral teeth). Teeth on the carapace extend along ~16% of the carapace length (CL) in the holotype (♂♂ 10–16%; ♀♀ 9–19% but may be as low as 5% in juvenile or subadult females: 5–10%). Supraorbital and antennal spines are present; pterygostomian angle is broadly rounded. Eyestalks are present but short and unpigmented.

The antennular peduncle (Fig. 2B) is 0.46× as long as the carapace (♂♂0.46–0.52; ♀♀ 0.38–0.47). The first segment is 1.67× as long as the second (♂♂1.64–1.84; ♀♀ 1.40–1.88), and the second segment is 1.27× longer than the third (♂♂1.27–1.76; ♀♀ 1.47–2.00). The first segment has an almost straight mesial margin, bearing sparsely distributed setose setae. The mesial margins of the second and third segments also carry a row of setose setae. Upper flagellum is uniramous, lower flagellum slender, lengths of both flagella exceed 200% of CL. Stylocerite has a slender, acute tip, reaching 0.83× the length of the basal segment of the antennular peduncle (♂♂ 0.83–0.98; ♀♀ 0.89–1.14). The distolateral lobe of the second segment is sharply pointed, its length being 24% of the peduncle length (♂♂ 22–25%; ♀♀ 21–33%). The antennal peduncle is 0.35× as long as the carapace (♂♂ 0.34–0.38; ♀♀ 0.23–0.31). Its scaphocerite (Fig. 2C) is 3.12× as long as wide (♂♂ 2.91–3.12; ♀♀ 2.73–3.36). The flagellum is mostly broken but exceeds at least 200% of CL.

**Abdominal somites, telson and uropods**. Abdominal somites (Fig. 2A) are smooth; total length of all six pleonites is 1.70× that of the carapace (♂♂ 1.59–1.80; ♀♀ 1.43–1.64). The sixth abdominal somite is 0.52× the length of the carapace (♂♂ 0.52–0.59; ♀♀ 0.50–0.53), 1.90× as long as the fifth somite (♂♂ 1.90–2.58; ♀♀ 1.78–2.27), and 1.05× as long as the telson (♂♂ 1.05–1.19; ♀♀ 1.12–1.15).

The telson (Fig. 2J, K) is 2.48× as long as it is wide proximally (♂♂ 2.48–2.60; ♀♀ 2.38–2.93); its proximal width is 1.55× greater than its distal width (♂♂ 1.44–1.59; ♀♀ 1.36–1.59). The lateral margins of the telson are slightly convex to straight, bear two pairs of dorsal spinules (♂♂ 2; ♀♀ 2) and one pair of dorsolateral spinules. The distal end is weakly bilobed and slightly concave medially. The distal end is armed with 12 spines (♂♂ 11–12; ♀♀ 12–13) situated between the distolateral spinules. Spine lengths are distinctly graded (from lateral to medial): the outermost, shortest dorsolateral spinule (1) is followed by the longest spine (1), typically at least 1.5× longer than any other along the margin; this is followed by a group of three (♂♂ 2–3; ♀♀ 3) intermediate-length spines that increase in size toward the centre, and finally by four (♂♂ 4; ♀♀ 3–4) centrally positioned spines, two on each side, that are the shortest overall, except for the distolateral spinules. The same pattern of spine length relationships was observed in other paratype males (adult TB379, subadult TB287); in TB380, which has only 13 spines (including the distolateral spinules), the shortest spine is still positioned distocentrally, although it appears solitary. A similar arrangement was also found in females.

The uropodal exopodite (Fig. 2D) is 3.08× as long as wide (♂♂ 3.08–3.69; ♀♀ 3.12–3.55), with the diaeresis (Fig. 2D) bearing one movable spinule (♂♂ 1; ♀♀ 1, exceptionally 2).

**Figure 3.**
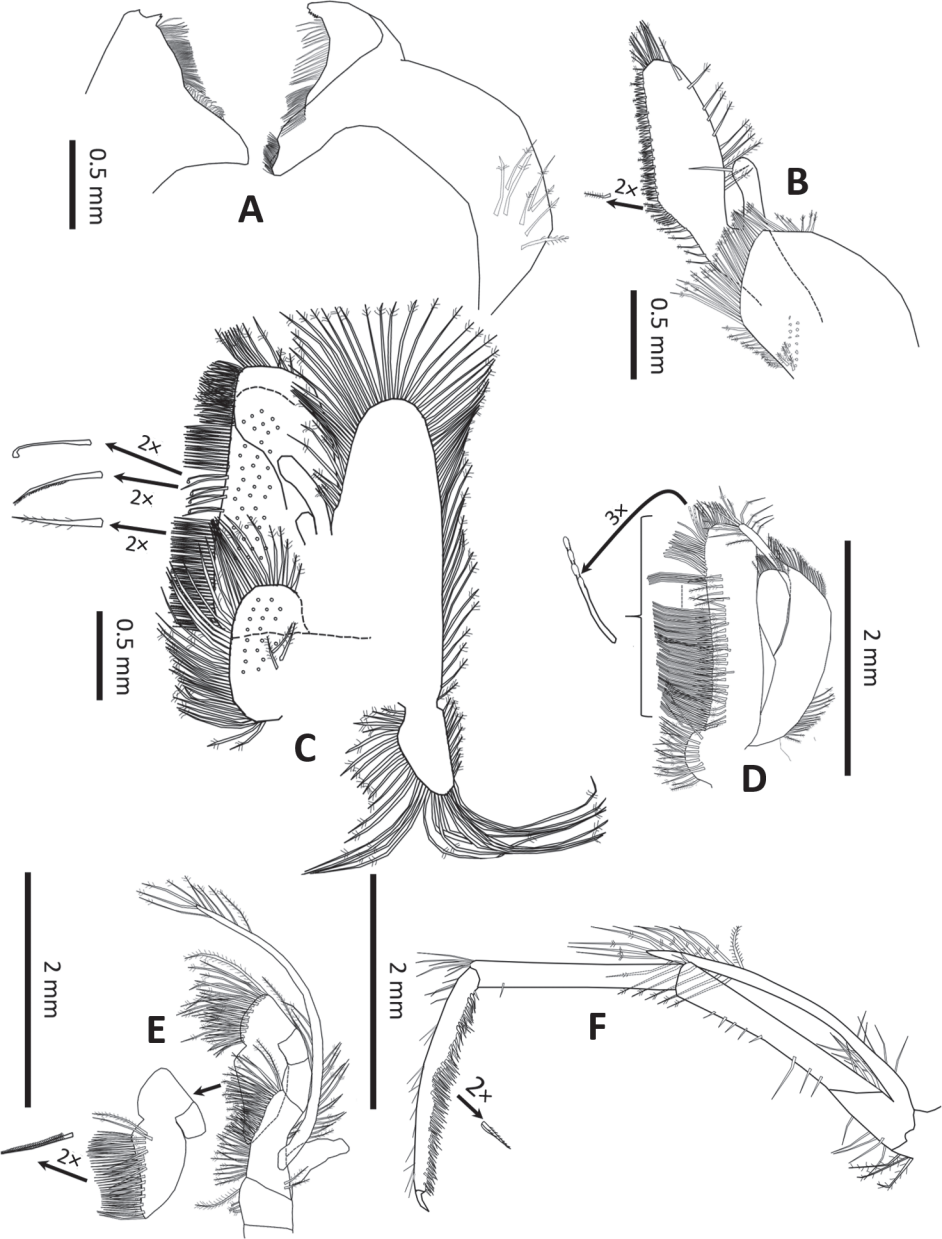
*Spelaeocaris electa* sp. nov. **A**. Mandible; **B**. Maxilla I; **C**. Maxilla II (all male, CL 7.0 mm, TB379, Plitica (cave), Dračevo, Popovo polje, Bosnia and Herzegovina); **D**. Maxilliped I; **E**. Maxilliped II; **F**. Maxilliped III (all holotype male, CL 6.6 mm, TB362, Buljovica (cave), Žakovo, Trebinje, Bosnia and Herzegovina).

***Mouthparts***. The mandible has a robust corpus and lacks a palp. Mandibular pars incisiva (incisor process; Fig. 3A) is stout and tapers distally, with a distal margin bearing 3–5 teeth of varying size. The number of teeth on the left and right mandibles is often different. Pars molaris is stout and U-shaped, with a triturative surface.

Maxilla I (Fig. 3B) palp is slender and truncated, bearing one long spiniform sub distal seta. The rectilinear outer margin of the upper lacinia (basipodial endite) is equipped with numerous short, strong cuspidate setae, interspersed with short plumose setae extending along the margin toward the coxal endite (6 setae in the holotype). The curvilinear inner margin bears a sparse row of plumose setae (11 in the holotype), with a cluster of plumose setae on the upper margin (10 in the holotype). The lower lacinia (coxal endite) is well developed and elongate, semi-circular in shape; its outer margin bears dense plumose setation, with an additional group of plumose setae present on the surface near the outer margin.

Maxilla II (Fig. 3C) has a slender, bilobed, tapering palp. The basipodial endite is also bilobed; the upper lobe is narrower (~1/5 the width of the lower lobe), with margins bearing dense plumose setae and the surface sparsely covered with plumose setae on both lobes. The coxal endite is fan-shaped, with dense plumose setae along the margin and sparsely distributed plumose setae on the surface. Scaphognathite is well developed, approximately as broad as the endite; its margin is fringed with plumose setae. The anterior lobe is large, while the posterior lobe is narrower, elongated, and subtriangular, with margins fringed by plumose setae. A group of more than 15 plumose setae on the lower distal part are the longest, with the longest being more than twice the length of the posterior lobe itself.

Maxilliped I (Fig. 3D) with a finger-like palp; the distal margin of the palp bears several plumose setae. The basipodial endite is ~3× longer than the coxal endite, with its distal margin bearing dense rows of long plumose setae and subequally long papulose setae with scale-like setules. The outer margin of the basipodial endite is almost rectilinear and bears a row of densely set plumose setae, while its mesial surface features a sparsely set row of plumose setae. The coxal endite is poorly developed, bearing plumose setae along its outer margin and a sparse distribution of plumose setae on its surface. The exopodite features a large caridean lobe, slightly more than twice as long as the flagellum. The lobe margin bears plumose setae at both proximal and distal ends, while the middle portion of the margin lacks setation. On the flagellum, only the distal part of the margin carries plumose setae. The surface of the lobe does not bear dense setation.

Maxilliped II (Fig. 3E) has a well-developed endopodite. The dactylopropodus is broad, bearing long plumose setae along the upper superior margin, densely packed longer pappose and shorter serrate setae along the nearly straight lower superior margin, and several long submarginal plumose setae. Exopodite has a well-developed flagellum, bearing plumose setae distally and serrate setae proximally. Podobranchium is well-developed, comb-like in structure, but lacking distinctly developed branches.

Maxilliped III (Fig. 3F) has a slender endopod, measuring 0.97× the carapace length (♂♂ 0.91–0.97; ♀♀ 0.88–1.08). The basis is poorly separated from the ischiomerus; together, the ischiomerus and basis are ~9× as long as wide; both bear a few sparse simple setae along their inferior margins. A few simple setae are present on the proximal part of the superior margin of the ischiomerus, with additional plumose setae along the inferior margin of the coxa. The penultimate segment is slender, ~9× as long as wide and subequal in length to the preceding ischiomerus, bearing several spiniform setae along the inferior margin and a group of longer and shorter simple setae on the disto-superior margin. The terminal segment (dactylopropodus) is ~10× as long as wide, slightly shorter than the penultimate segment and equal in length to the ischiomerus. It tapers distally and bears a strong apical dactylopropodal spine (claw). The mesial surface bears dense serrate setation, while the upper margin has sparse simple setae. The exopodite tip extends beyond the distal end of the basioischiomere, with a robust flagellum bearing long distal plumose setae. The coxa carries a well-developed arthrobranchium (frequently damaged during dissection).

**Figure 4.**
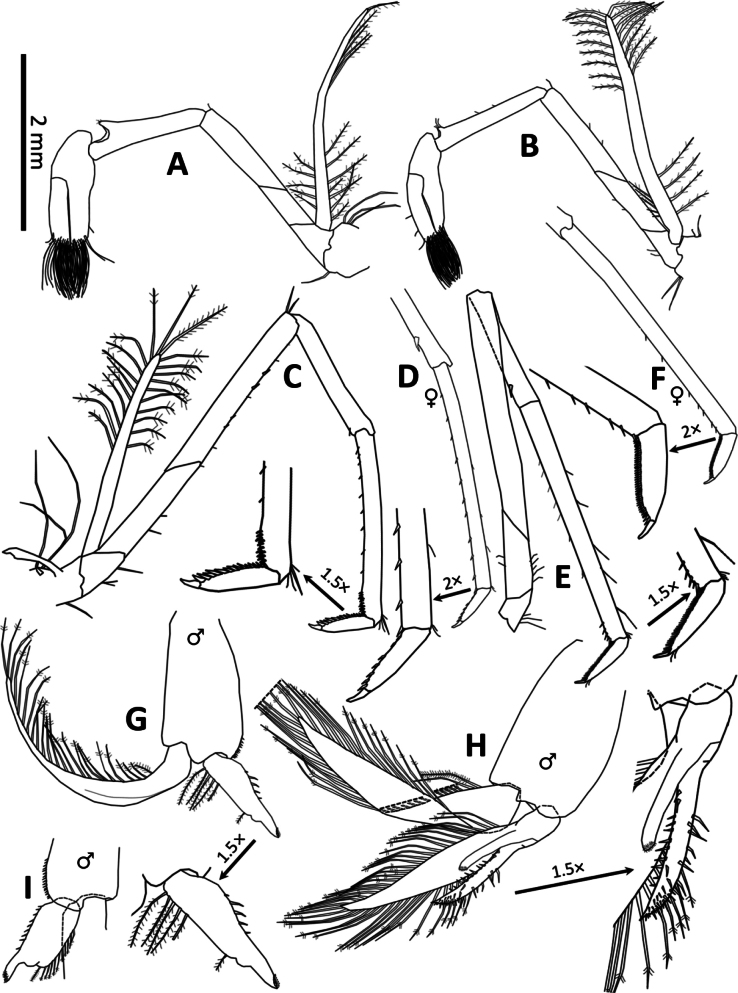
*Spelaeocaris electa* sp. nov. **A**. Pereiopod I; **B**. Pereiopod II; **C, D**. Pereiopod III; **E, F**. Pereiopod V (♀ – female; not designated – male); **G**. Pleopod I (♂ – male); **H**. Pleopod II (♂ – male); **I**. Pleopod I endopodite (♂ – male). Except for (**D, F** and **I**), all drawings represent holotype male, CL 6.6 mm, TB362, Buljovica (cave), Žakovo, Trebinje, Bosnia and Herzegovina; (**D, F)** female, CL 6.6 mm, TB361, data same as holotype and (**I**) adult male, CL 6.4 mm, TB380, Plitica (cave), Dračevo, Popovo polje, Bosnia and Herzegovina.

***Pereiopods***. The first pereiopod (Fig. 4A), measured from the base of segment 2 (basis) to the apex of segment 6 (propodus, unmovable finger of the chela), is 0.68× the length of the carapace (♂♂ 0.65–0.79; ♀♀ 0.66–0.79). The chela and carpus of the first pereiopod are stouter and broader than those of the second pereiopod (Fig. 4B). The maximum length of the chela of the first pereiopod is 2.87× (♂♂ 2.87–3.31; ♀♀ 2.69–3.82) its width. The chela is approximately the same length as (in the holotype) or slightly shorter than the carpus (♂♂ 0.86–1.01; ♀♀ 0.89–1.10). The chela has a subcylindrical, slightly compressed palm; the fingers bear a dense tuft of longer plumose and shorter serrate setae apically, with rounded tips. The dactylus is 60% (♂♂ 60–63; ♀♀ 57–71) of the propodus’ maximum length. The carpus is well excavated distally and is 1.28× (♂♂ 1.28–1.53; ♀♀ 1.26–1.74) the length of the merus. The merus is 1.84× (♂♂ 1.33–1.84; ♀♀ 1.04–1.76) longer than the ischium, with the basis being the shortest segment of the pereiopods. The first pereiopod has a well-developed exopodite that extends well beyond the fourth segment (merus, almost reaching the distal end of the carpus) and bears numerous plumose setae along the proximal and distal parts of its margin.

**Figure 5.**
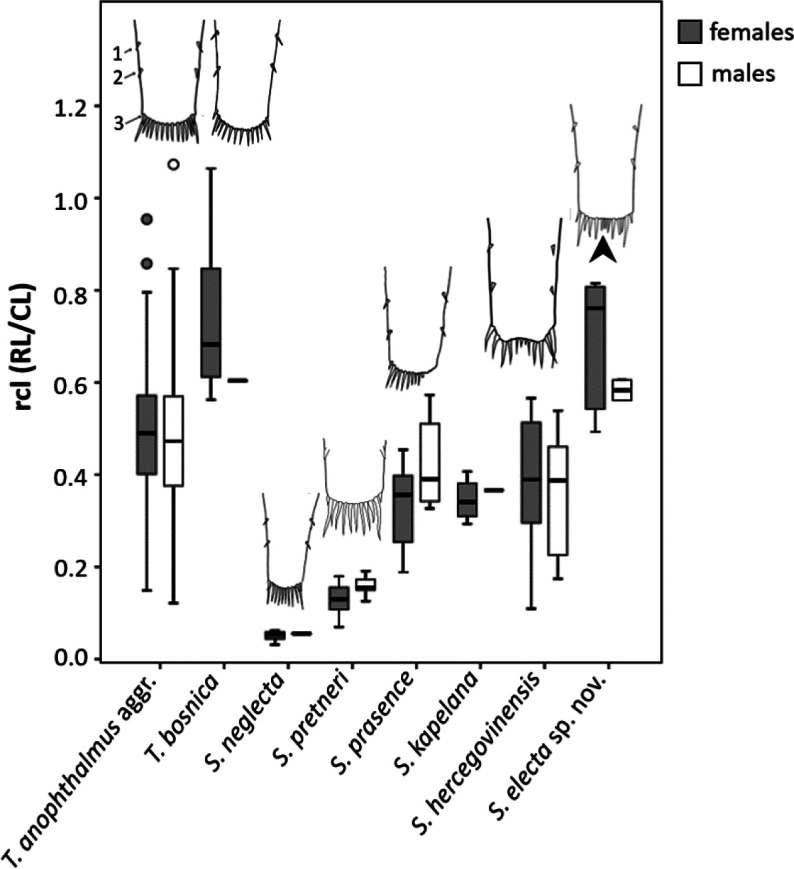
Variation in relative rostral length (rcl) and telson morphology in eight taxa of the genera *Troglocaris* and *Spelaeocaris*. Number of measured females/males: *T. anophthalmus* aggr. (= *T. anophthalmus* spp. + *T. planinensis*) – 315/165; *T. bosnica* – 12/1; *S. neglecta* – 8/2; *S. pretneri* – 25/7; *S. prasence* – 15/6; *S. kapelana* – 4/1; *S. hercegovinensis* – 17/11 (dataset from Jugovic et al. 2011); *Spelaeocaris electa* sp. nov. – 8/2 (present study). Telson sketches adapted and redrawn from: *T. anophthalmus* aggr. (Jugovic et al. 2019); *T. bosnica*, *S. neglecta*, *S. prasence* (Sket and Zakšek 2009); *S. pretneri* (Matjašič 1956); *S. hercegovinensis* (Babić 1922). Note the slightly concave posterior margin of the telson in the two sister species: *S. hercegovinensis* and *Spelaeocaris electa* sp. nov. (indicated by short black arrow), in contrast to the convex distal margin in all other species (sketch for *S. kapelana* is not provided but its distal margin is convex (see Sket and Zakšek 2009)). Thin arrows with numbers 1, 2 and 3 on the upper left sketch denote dorsomarginal (1–3) spiniform setae, of which the pair 3 is dorsomarginal-terminal. Abbreviations: RL – rostral length, CL – carapace length.

The second pereiopod (Fig. 4B), measured from the base of segment 2 (basis) to the apex of segment 6 (propodus, the fixed finger of the chela), is 0.74× (♂♂ 0.74–1.02; ♀♀ 0.86–0.98) the length of the carapace. Maximal length of the chela of the second pereiopod is 3.79× (♂♂ 3.06–3.79; ♀♀ 3.09–4.05) as long as wide, and 0.92× (♂♂ 0.70–0.92; ♀♀ 0.67–0.75) the length of the carpus; the chela has a subcylindrical, slightly compressed palm, with fingers bearing dense tufts of longer pappose and shorter serrate setae apically, and rounded tips. The dactylus is 55% (♂♂ 55–72; ♀♀ 60–73) of the maximal length of the propodus; the carpus is well excavated distally (though less so than in the first pereiopod), measuring 1.11× (♂♂ 1.11–1.45; ♀♀ 1.26–1.55) the length of the merus. Merus is 1.60× (♂♂ 1.43–2.17; ♀♀ 1.04–1.81) longer than the ischium, with the basis being the shortest segment of the pereiopods. The second pereiopod has a well-developed exopodite that extends well beyond segment 4 (merus), reaching approximately the middle of the carpus, and bears numerous plumose setae along the proximal and distal parts of its margin.

The third pereiopod is slender (Fig. 4C, D), with a length from the base of segment 2 (basis) to the apex of segment 7 (dactylus) measuring 1.25× (♂♂ 1.25–1.54; ♀♀ 1.36–1.58) the length of the carapace. The dactylus terminates in a large claw with 15 (♂♂ 15; ♀♀ 7–8) accessory spines on the flexor margin; the propodus is 2.91× (♂♂ 2.91–4.39; ♀♀ 3.91–4.87) as long as the dactylus; the carpus is 0.66× (♂♂ 0.60–0.68; ♀♀ 0.54–0.61) as long as the propodus and 0.58× (♂♂ 0.58–0.61; ♀♀ 0.52–0.61) as long as the merus; the merus is 1.71× (♂♂ 1.63–1.71; ♀♀ 1.64–1.91) as long as the carpus and 2.72× (♂♂ 2.23–2.72; ♀♀ 2.18–3.62) as long as the ischium. The ischium is always longer than the basis, which is the shortest segment of the third pereiopod. In adult males, the propodus (segment 6) is distally widened, with the enlargement occurring at the distal 15% (♂♂ range: 9–15; ♀♀ undifferentiated) of its length. The maximal width of the propodus—measured at its widest point—represents 10% (♂♂ 6–10; ♀♀ 5–7) of the propodus length and 65% (♂♂ 65–79) of the differentiated distal part (as measured above). In adult males (and to a lesser extent in subadults), the distal enlargement of the propodus bears a dense group of ~12 (♂♂ 9–18; ♀♀ without a group of spiniform setae) spiniform setae. Additionally, along the inferior margin of the propodus before the enlargement, there is a sparse set of 3 (♂♂ 3–14; ♀♀ 9–14) spiniform setae, which are sparsely distributed along the inferior margin of propodus before the enlargement. Carpus, merus and ischium with 0 (♂♂ 0–1; ♀♀ 0–1), 4 (♂♂ 4–5; ♀♀ 3–5) and 0 (♂♂ 0–1; ♀♀ 0–1) spiniform setae, respectively. The basis lacks spiniform setae. The third pereiopod has a well-developed exopodite reaching approximately to the middle of the fourth segment (merus) and bears sparsely distributed plumose setae along its distal half.

The fourth pereiopod is similar to the third pereiopod, with a length from the base of segment 2 (basis) to the apex of segment 7 (dactylus) measuring 1.42× the carapace length (♂♂ 1.37–1.45; ♀♀ 1.28–1.46). The dactylus terminates in a single large claw, with 19 accessory spines on the flexor margin (♂♂ 11–19; ♀♀ 7–9). The propodus is 3.80× as long as the dactylus (♂♂ 3.69–4.01; ♀♀ 4.05–5.48). The carpus is 0.58× as long as the propodus (♂♂ 0.58–0.65; ♀♀ 0.43–0.55) and 0.63× as long as the merus (♂♂ 0.62–0.63; ♀♀ 0.53–0.63). The merus is 1.58× as long as the carpus (♂♂ 1.58–1.60; ♀♀ 1.59–1.90), and 2.12× as long as the ischium (♂♂ 2.12–3.38; ♀♀ 1.94–2.83). The ischium is always longer than the basis, which is the shortest segment of the fourth pereiopod. In adult males, the propodus (segment 6) is distally widened, with the enlargement occurring at the distal 12% of its length (♂♂ 11–14; ♀♀ undifferentiated). The maximal width of the propodus (measured at its widest point) represents ~9% of the propodus length (♂♂ 8–9; ♀♀ 5–9) and 75% (♂♂ 60–75) of the differentiated distal part (as measured above). In adult males (but also noticed in a smaller extent in subadults), the distal enlargement contains a dense group of ~14 (♂♂ 10–17; ♀♀ without a group of spiniform setae) spiniform setae and bears a sparse series of eight (♂♂ 7–11; ♀♀ 8–12 sparse spiniform setae along the whole length of propodus) spiniform setae along the inferior margin of the propodus before the enlargement. The carpus, merus, and ischium bear 0 (♂♂ 0; ♀♀ 0–1), 3 (♂♂ 3–4; ♀♀ 3), and 0 (♂♂ 0–1; ♀♀ 0–1) spiniform setae, respectively. The basis lacks spiniform setae. The fourth pereiopod possesses a well-developed exopodite that reaches the midpoint of segment 4 (merus) and bears numerous plumose setae along the proximal and distal portions of its margin.

The fifth pereiopod (Fig. 4E, F) is slender, measuring 1.36× the carapace length from the base of segment 2 (basis) to the apex of segment 7 (dactylus) (♂♂ 1.31–1.52; ♀♀ 1.34–1.55). The dactylus terminates in a single large claw, bearing a series of 44 spiniform setae (♂♂ 44–46; ♀♀ 42–48) that form a comb along the flexor margin. The propodus is 5.11× as long as the dactylus (♂♂ 5.11–5.38; ♀♀ 4.68–5.18). The carpus is 0.47× the length of the propodus (♂♂ 0.47–0.49; ♀♀ 0.43–0.48), and 0.58× the length of the merus (♂♂ 0.57–0.64; ♀♀ 0.58–0.61). The merus is 1.73× as long as the carpus (♂♂ 1.56–1.75; ♀♀ 1.63–1.74), and 2.17× as long as the ischium (♂♂ 1.82–3.18; ♀♀ 1.77–2.13). The ischium is consistently longer than the basis, which is the shortest segment of the fifth pereiopod. The propodus and carpus bear 8 (♂♂ 6–8; ♀♀ 4–12) and 1 (♂♂ 1; ♀♀ 0–1) spiniform setae, respectively. The merus, ischium, and basis bear no spiniform setae, with the exception of a single female specimen bearing two spines on the merus. The fifth pereiopod does not have an exopodite and lacks a well-developed distal enlargement (or widening) of the distal portion of the propodus.

Pereiopods I–IV each bear a pleurobranchium, epipodite, and exopodite. Pereiopod V bears a pleurobranchium but lacks both an epipodite and exopodite. All pereiopods possess setobranchia.

***Pleopods***. The endopodite of the male first pleopod (Fig. 4G) consists of an oval to somewhat lobate lamina that extends into a finger-like appendix interna (9-shaped sensu Sket and Zakšek 2009), measuring 0.40× the length of the exopodite (♂♂ 0.37–0.42). The appendix interna bears a distal group of ~25 (~12–~25) retinacular hooks. The inner margin of the endopodite bears a sparse row of simple setae (~5–9), while the outer margin carries a sparsely arranged row of longer plumose setae (~7–11). The margins of the exopodite are fringed with long, evenly distributed plumulose setae.

The appendix masculina of the male second pleopod (Fig. 4H) is slender and spindle-shaped, measuring 0.75× the length of the endopodite (♂♂ 0.61–0.75) and 2.19× the length of the appendix interna (♂♂ 1.85–2.57). The appendix masculina bears numerous spiniform setae distributed fairly evenly across its surface, with a few of the longest setae exceeding the width of the appendix interna (in specimen TB380, the longest setae extend to at least three-quarters of the width of the appendix masculina). The appendix interna is ~1/2 as slender as the appendix masculina and bears ~30 retinacular hooks at its distal end (♂♂ ~20–30). The exopodite is 1.34× (range 1.15–1.34) longer than the endopodite. The distal part of the protopodite bears a series of ~11–16 short spiniform setae along the inner margin. Both the exopodite and endopodite margins are fringed with long, evenly distributed plumulose setae.

The endopodite of the female first pleopod (Fig. 2G, H) is slender and distally elongated, measuring 0.82–0.91× the length of the exopodite. The distal elongation corresponds to the appendix interna but lacks retinacular hooks at its tip. Along the outer margin of the endopodite, there is a sparse series of plumulose setae (2–19). Variability in the shape of the endopodite is clearly visible (see Fig. 2G, H); the distal part of the endopodite can be either weakly or markedly elongated, and in the latter case, it is distinctly narrower than the proximal part. In females with a notably elongated endopodite, the distal half of its inner margin bears a series of plumose setae (0–15). The female second pleopod (Fig. 2I resembles that of the male but lacks an appendix masculina; its exopodite is 1.11–1.21× as long as the endopodite, and the appendix interna is 0.28–0.32× as long as the endopodite.

##### Sexual dimorphism and geographical variability.

Males and females are approximately the same length and cannot be treated as sexually dimorphic in size with present data (SDI = -0.08). We also noticed sexual dimorphism in pleopods 1 and 2 (as described above). A significant difference was recorded in number of accessory spines on the flexor margins of dactylus III (males: ~15; females: 6–8) and IV (males: 11–19; females: 7–9), but not V (males: 44–46; females: 41–48). Antennae I–II are apparently relatively longer (in relation to CL) in males than in females (♂♂ mean ± SE – A I (*n* = 4): 0.48 ± 0.014; A II (*n* = 4): 0.35 ± 0.009, ♀♀ mean ± SE – A I (*n* = 4): 0.43 ± 0.009; A II (*n* = 5): 0.29 ± 0.014, One-way T-test for AI and AII, p < 0.01), but larger sample would be needed to precisely ascertain this and some other sexual differences (see also Suppl. material 3). Two geographically most distant individuals (the northern two locations) have different haplotypes and show some genetic distance to the individuals from southern locations although no differences in morphological characters were measured (on a very limited number of samples).

##### Reproductive biology.

Although no ovigerous females were present in our sample and no information on egg size and volume can be given, pleopods of one female (TB378) bore multiple long setae (see Fig. 2G), i.e. probably “soies ovigères” (egg-bearing setae). “Soies ovigères” should develop before females paste their eggs onto the elongated setae on pleopods I–III and remain until the first moult even after hatching (Juberthie-Jupeau 1975; Jugovic et al. 2010b). Most probably, this female carried no eggs hence it was probably caught in a later described stage.

##### Etymology.

The species epithet *electa* means ”candidate, the chosen one” and it reflects the specific procedure of its name selection. This name was selected among the three candidate names, by public online voting via social media and voting of participants of the 3^rd^ Dinaric Symposium on Subterranean Biology in Trebinje (Bosnia and Herzegovina). This voting was organized as part of the raising public awareness activities, within the project “SubBioCODE: Developing new tools for rapid assessment of subterranean biodiversity in Bosnia and Hercegovina”, conducted between 2019–2022 by the University of Ljubljana (Slovenia) and financially supported by the foundation Critical Ecosystems Partnership Fund (CEPF).

##### Distribution.

**Figure 6.**
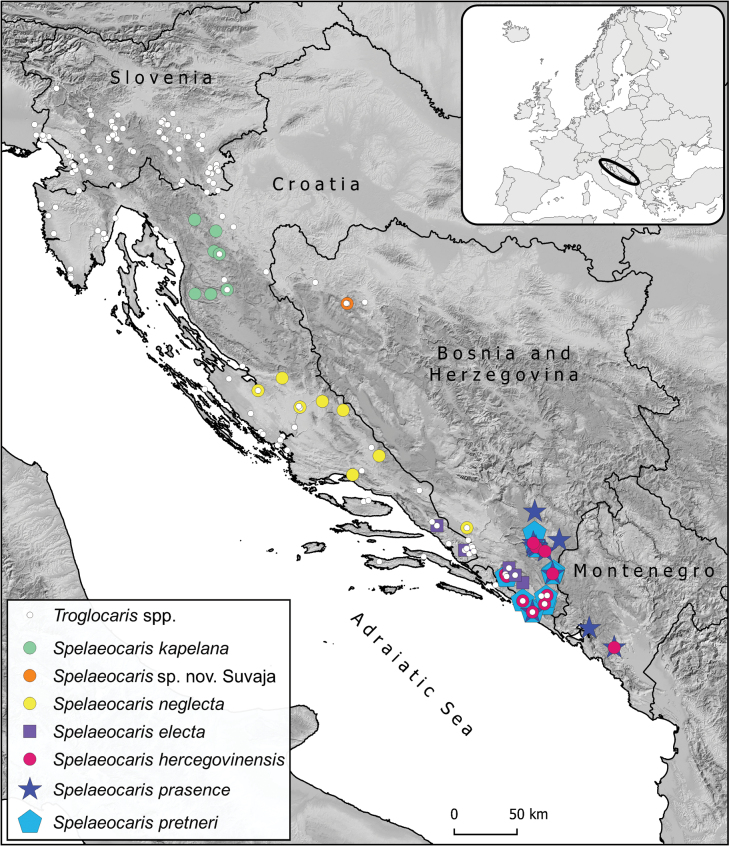
Distribution of *Spelaeocaris electa* sp. nov. and other *Spelaeocaris* species: *S. hercegovinensis*, *S. prasence*, *S. pretneri*, *S. neglecta*, *S. kapelana* and *S.* sp. nov. Suvaja on the Dinaric Karst. In addition, distribution of *Troglocaris* spp. is shown with small white circles. Distributional data derived from material collected for this study (Suppl. material 1) and from SubBioDB, database on subterranean biodiversity managed by SubBioLab at University of Ljubljana, which includes literature and collected material data.

*Spelaeocaris electa* sp. nov. was found at five localities (caves) in a narrow belt along the Adriatic coast of southern Dalmatia, namely in two caves in southeastern Croatia and in three caves in the northeastern part of Popovo polje (Bosnia and Herzegovina) (Fig. 6, Suppl. material 1). All known localities access subterranean waters of the Neretva River basin, more specifically, the lower Neretva River and the Trebišnjica River. The distribution range (derived from Fig. 6) extends ~80 km in linear distance between the geographically most distant localities, which is similar to range sizes of other cave shrimps in southeastern Dinaric Karst, where the linear distance between the two most distant localities ranges from 60 km (*S. pretneri*) to ~100 km (*S. prasence* and *S. hercegovinensis*).

##### Remarks.

Two genera of cave shrimps are known from the Dinaric Karst: *Troglocaris*, distributed from northeastern Italy to southern Bosnia and Herzegovina, and *Spelaeocaris*, distributed from Croatia to Montenegro (Fig. 6). In all five surveyed caves, the new species was found co-occurring with *Troglocaris anophthalmus periadriatica*. In one of these caves, Plitica (cave) near Dračevo, a single individual of the third species, *S. prasence*, was also recorded (voucher TB376). Based on the data in hand, the sister species *Spelaeocaris electa* sp. nov. and *S. hercegovinensis* have allopatric distributions (Fig. 6).

## Discussion

### Morphology and diversity of European atyid shrimps

**Figure 7.**
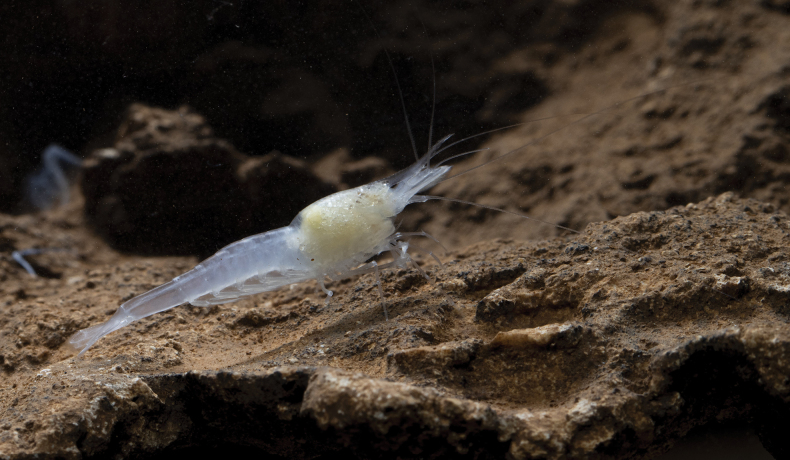
Photo of the cave shrimp *Spelaeocaris electa* sp. nov. from Plitica (cave) near Dračevo in Bosnia and Herzegovina. Photo credit: TD.

In comparison with *Spelaeocaris electa* sp. nov. (Fig. 7), the closely related cave shrimp species from the genus *Xiphocaridinella* from Caucasus have in most cases a shorter and predominantly toothless rostrum (see Marin and Barjadze 2022 and references therein). All *Xiphocaridinella* species have a convex distal margin of the telson, while *Spelaeocaris electa* sp. nov. exhibits a slightly bilobed telson margin. The arrangement of spines along the distal margin of the telson (see Figs 2, 5for relative spine lengths) is characteristic of *Spelaeocaris electa* sp. nov., and, together with the length of the rostrum, serves to distinguish it from all other *Spelaeocaris* species. Although rostral length is one of the discriminative traits between *Spelaeocaris electa* sp. nov. and its sister species, *S. hercegovinensis*, it is known to be very variable in *Troglocaris* and should be used with caution in taxonomy of atyids (e.g. Jugovic et al. 2010b; Mazancourt et al. 2017). A detailed comparison of morphology with other cave shrimps from the Dinaric Karst is included in Suppl. material 3 and Suppl. material 4.

*Spelaeocaris electa* sp. nov. differs from other known European atyid shrimps found outside the Dinaric Karst by a combination of diagnostic characters discussed above. The non-Dinaric (although on the Balkan Peninsula) atyid cave shrimp *Ficticaris serbica* Jugovic and Sket 2019, endemic to eastern Serbia (Jugovic et al. 2019), differs by having a short, toothless rostrum that slightly overreaches the eye rudiments, and a strongly bilobed distal margin of a telson (which is much more pronounced than in *Spelaeocaris electa* sp. nov.). In addition, *F. serbica* also has maxilliped III without flagellum (Jugovic et al. 2019). Another cave species, *Gallocaris inermis* (Fage, 1937) from southern France (Sket and Zakšek 2009), has maxilliped III with a short flagellum (< width of lobe; in *Spelaeocaris electa* sp. nov. it is longer than its width) and pereiopod V dactylus not comb-like. All other atyid shrimp species from Europe and northern Africa are epigean, i.e. with body pigments and/or with well-developed and pigmented eyes.

### Implications for conservation

Cave shrimps of the Balkan Peninsula have gained research attention for over a century, with several new species and subspecies described in recent years, particularly from the Dinaric Karst (Sket and Zakšek 2009; Jugovic et al. 2012, 2019). Collected data show that most localities are inhabited by a single cave shrimp species (SubBioDB 2025); however, in the southeastern part of Dinarides, species rich caves (Culver and Sket 2000), hosting more than a single species, are known. The richest, the Vjetrenica cave system, renowned for its exceptionally rich subterranean biodiversity (Delić et al. 2023), was long known as the only one hosting three cave shrimp species: *Spelaeocaris hercegovinensis*, *S. pretneri* and *Troglocaris anophthalmus periadriatica* (Matjašič 1960).

To date, four species of atyids are reported from the southern Dinaric Karst: *T. a. periadriatica*, *S. hercegovinensis*, *S. pretneri* and *S. prasence* (Sket and Zakšek 2009; Jugovic et al. 2012). Recent findings of *S. neglecta* in Vrelo Vakuf (Studenci, Bosnia and Herzegovina; Fig. 6, Suppl. material 1) extend the known range of this species further south and, with a description of *Spelaeocaris electa* sp. nov., raises the number of Herzegovinian species to six. Given the already high diversity, the discovery of a new species was somewhat unexpected. It was initially revealed through molecular analyses and further supported by detailed morphological studies. This finding highlights the lack of comprehensive speleobiological surveys in this area and shows that even in a renowned global hotspot of subterranean biodiversity like the Dinaric Karst, new species continue to be discovered (e.g. Delić et al. 2017).

Despite recent taxonomic advances and descriptions, the list of European cave shrimps is still not complete. At least one undescribed species of *Spelaeocaris* is still hidden in less explored karstic areas of Bosanska Krajina (Figs 1, 6). This species, referred to as the “Bosnian clade” (Sket and Zakšek 2009), known from a single female collected in Suvaja pećina (*Spelaeocaris* sp. Suvaja on Fig. 1), needs additional specimens that would enable its formal description. Furthermore, given the high species richness of cave shrimps in the southern Dinaric Karst, their co-occurrence and their limited distribution, it is likely that this region harbours additional yet undescribed taxa.

The discovery and formal description of a narrowly distributed, endemic cave shrimp species is not only of scientific importance but also of immense conservation value, as species with small geographical ranges are particularly vulnerable to extinction (Niemiller et al. 2013). This is especially important in areas such as the southern Dinaric Karst, where increasing infrastructural development (e.g. hydropower plants network) and economic pressures pose serious threats to natural ecosystems (Fišer et al. 2022).

### The underexploited outreach of taxonomy

The finding of a new cave shrimp also provided a valuable opportunity for public participation in the species naming process through a public voting campaign. Our initiative successfully engaged participants through a combination of digital media (Facebook polling) and physical (conference ballot) voting, allowing a participatory approach in the selection of the species’ specific epithet. The entire initiative was unexpectedly augmented with local media, including regional radio and television coverage, which broadcasted conference and acquainted locals with the existence of newly discovered species. These results indicate that incorporating public participation into traditionally academic-limited processes, such as taxonomic nomenclature, has the potential to serve as a tool for science communication and may further enhance public interest in biodiversity and its conservation.

The Dinaric Karst is a global hotspot of subterranean groundwater biodiversity, harbouring many local endemics. Therefore, raising awareness of local residents is essential for safeguarding natural resources. The fauna of the Dinaric Karst is highly threatened by planned hydro-engineering developments (Fišer et al. 2022), and local initiatives are often the only means of influencing local decision-making processes. In this context, species-naming campaigns can serve as an additional tool to raise local awareness and contribute to biodiversity conservation.

## Supplementary Material

XML Treatment for Spelaeocaris electa

## Additional information

### Use of AI

No use of AI was reported.

### Author ORCIDs

Jure Jugovic https://orcid.org/0000-0002-1963-1975

Cene Fišer https://orcid.org/0000-0003-1982-8724

Maja Zagmajster https://orcid.org/0000-0003-1323-9937

Teo Delić https://orcid.org/0000-0003-4378-5269

Valerija Zakšek https://orcid.org/0000-0002-6503-7424

### Data availability

All of the data that support the findings of this study are available in the main text or Supplementary Information.

## Supplementary materials

Supplementary material 1 List of samples Data type xlsx Explanation note List of samples used in phylogenetic analyses along with: vouchers, occurrences, sequences (GenBank Accession numbers). https://binary.pensoft.net/file/1517117This dataset is made available under the Open Database License (http://opendatacommons.org/licenses/odbl/1.0/). The Open Database License (ODbL) is a license agreement intended to allow users to freely share, modify, and use this Dataset while maintaining this same freedom for others, provided that the original source and author(s) are credited. Jure Jugovic, Cene Fišer, Maja Zagmajster, Teo Delić, Valerija Zakšek

Supplementary material 2 PCR protocols, data partitioning and models, image, genetic distances Data type docx Explanation note PCR protocols, dataset partitioning and models used in partitions; voting card used for species name selection on the 3^rd^ Dinaric Symposium on Subterranean Biology, held in Trebinje (Bosnia and Herzegovina) in April 2022; estimates of pairwise genetic distances under Kimura 2-parameter distance model (K2P); in % differences for the cytochrome oxidase I (COI) gene sequences between seven *Spelaeocaris* species. https://binary.pensoft.net/file/1517118This dataset is made available under the Open Database License (http://opendatacommons.org/licenses/odbl/1.0/). The Open Database License (ODbL) is a license agreement intended to allow users to freely share, modify, and use this Dataset while maintaining this same freedom for others, provided that the original source and author(s) are credited. Jure Jugovic, Cene Fišer, Maja Zagmajster, Teo Delić, Valerija Zakšek

Supplementary material 3 Morphological data Data type docx Explanation note Comparison of *Spelaeocaris electa* sp. nov. with its sister species *S. hercegovinensis*, other species of *Spelaeocaris* = *S. pretneri* + *S. neglecta* + *S. kapelana* + *S. presence* and genus *Troglocaris* = *T. anophthalmus*, *T. planinensis* and *T. bosnica*. https://binary.pensoft.net/file/1517119This dataset is made available under the Open Database License (http://opendatacommons.org/licenses/odbl/1.0/). The Open Database License (ODbL) is a license agreement intended to allow users to freely share, modify, and use this Dataset while maintaining this same freedom for others, provided that the original source and author(s) are credited. Jure Jugovic, Cene Fišer, Maja Zagmajster, Teo Delić, Valerija Zakšek

Supplementary material 4 Detailed comparison of *Spelaeocaris electa* sp. nov. Data type docx Explanation note Detailed comparison of *Spelaeocaris electa* sp. nov. with its sister species *S. hercegovinensis* and other species of *Spelaeocaris* and *Troglocaris*. https://binary.pensoft.net/file/1517120This dataset is made available under the Open Database License (http://opendatacommons.org/licenses/odbl/1.0/). The Open Database License (ODbL) is a license agreement intended to allow users to freely share, modify, and use this Dataset while maintaining this same freedom for others, provided that the original source and author(s) are credited. Jure Jugovic, Cene Fišer, Maja Zagmajster, Teo Delić, Valerija Zakšek
