# Supplementary material for: Revealing European cave shrimp diversity: a new species of Spelaeocaris (Decapoda, Atyidae) named through public participation
Source: Zookeys. 2026 Jan 22;1267:51–76. doi: 10.3897/zookeys.1267.176622 (PMC12856486; doi:10.3897/zookeys.1267.176622)
Supplement: Supplementary material 2 — PCR protocols, data partitioning and models, image, genetic distances [file zookeys-1267-051_article-176622__-s002.docx]

**Supplementary Material 2**

*PCR protocols*, *dataset partitioning and models used in partitions*

PCR protocols used for amplification of each fragment.

| **COI** |  | **16S** |  | **ITS2** |  |
| --- | --- | --- | --- | --- | --- |
| 94°C for 3 min |  | 94°C for 3 min |  | 94 °C for 3 min |  |
| 94°C for 45 sec |  | 94°C for 30 sec |  | 94 °C for 45 sec |  |
| 48°C for 45 sec | 34 x | 47°C for 30 sec | 40 x | 48 °C for 45 sec | 34 x |
| 72°C for 1 min |  | 72°C for 45 sec |  | 72 °C for 1 min 30 sec |  |
| 72°C for 3 min |  | 72°C for 10 min |  | 72 °C for 3 min |  |

DNA partitions and partitions with specific optimal substitution models as selected using the program PartitionFinder v.2.1 and were used in phylogenetic analyses.

| **Gene** | **Primer pair** | **Partition** | **Best Substitution model** | **Reference** |
| --- | --- | --- | --- | --- |
| COI | LCO1490 – HCO2198 | 1-642/3  2-642/3 | TIM+I  F81+I | Folmer *et al.*, 1994 |
|  |  | 3-642/3 | GTR+I+G |  |
|  |  |  |  |  |
| 16S rDNA | 16Sar – 16Sbr | 643-1125 | GTR+I+G | Simon *et al.* 1991 |
|  |  |  |  |  |
| ITS2 | ITS3-ITSVIIIrev | 1126-2223 | GTR+I+G | White *et al.* 1990, Zakšek *et al.* 2009 |

**References:**

Folmer O.M., Hoeh Black M., Lutz R., Vrijehoek R. 1994. DNA primers for amplification of mitochondrial cytochrome c oxidase subunit I from diverse metazoan invertebrates. Mol. Mar. Biol. Biotechnol., 5, pp. 304-313.

Simon C., Franke A., Martin A. 1991. The polymerase chain reaction: DNA extraction and amplification. G.M. Hewitt, A.W.B. Johnson, J.P.W. Young (Eds.), Molecular Techniques in Taxonomy, Springer Verlag, Berlin, pp. 329-355.

White T.J., Bruns T., Lee S., Taylor J. 1990. Amplification and direct sequencing of fungal ribosomal RNA genes for phylogenetics M.A. Innis, G.H. Gelfand, J.J. Sninsky, T.J. White (Eds.), PCR Protocols, Academic Press, San Diego, pp. 315-332.

# Zakšek V., Sket B., Gottstein S., Franjević D., Trontelj P. 2009. The limits of cryptic diversity in groundwater: phylogeography of the cave shrimp *Troglocaris anophthalmus* (Crustacea: Decapoda: Atyidae). Molecular Ecology 18: 931-946.

**Figure S1**

**
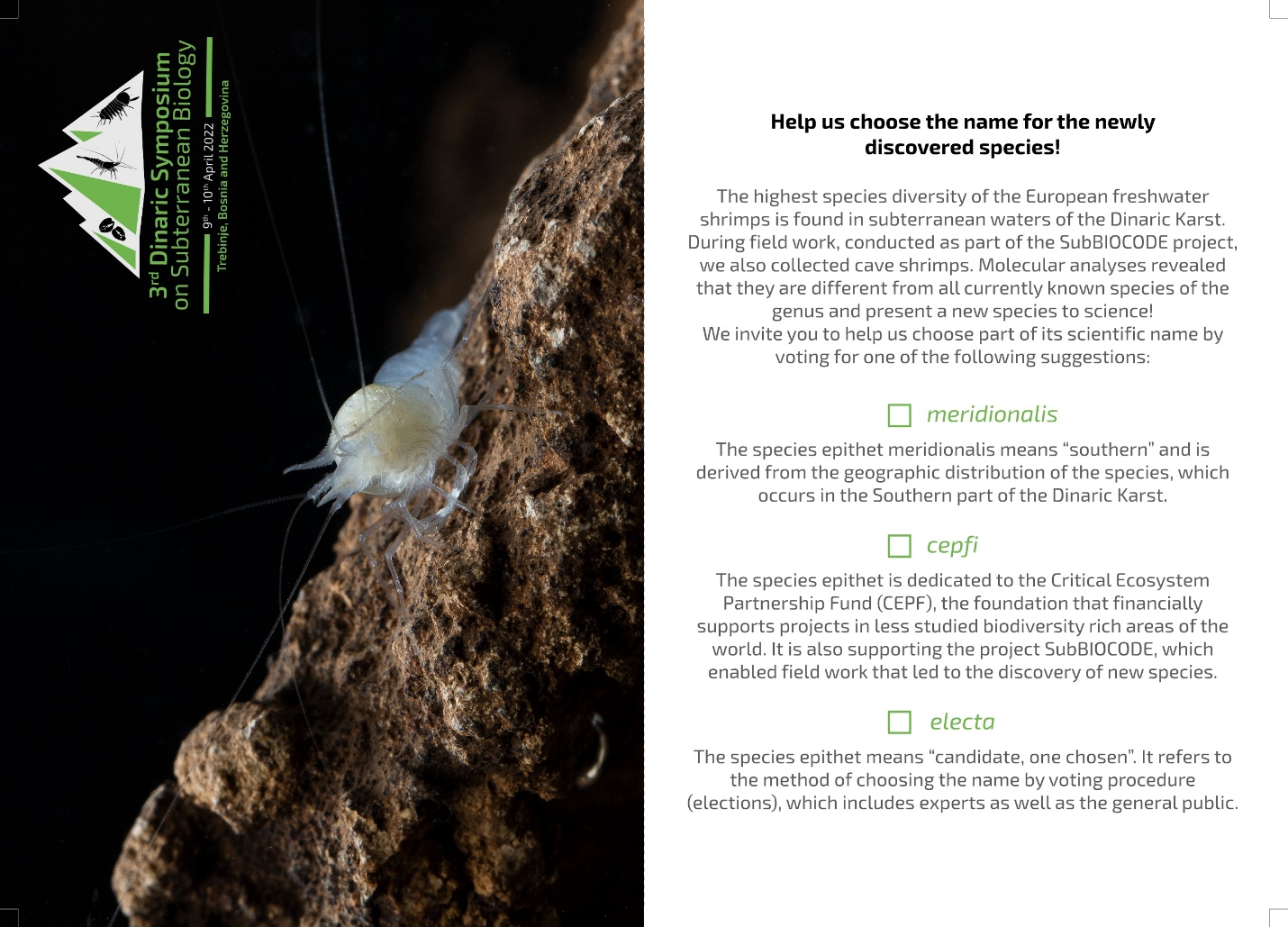
**

**Figure S1.** Voting card used for species name selection on the 3^rd^ Dinaric Symposium on Subterranean Biology, held in Trebinje (Bosnia and Herzegovina) in April 2022.

**Table S1.** Estimates of pairwise genetic distances under Kimura 2-parameter distance model (K2P); in % differences for the cytochrome oxidase I (COI) gene sequences between seven *Spelaeocaris* species.

|  | ***S. Suvaja 3*** | ***S. hercegovinensis*** | ***S. pretneri*** | ***S. prasence*** | ***S. neglecta*** | ***S. kapelana*** |
| --- | --- | --- | --- | --- | --- | --- |
| ***S. Suvaja 3*** |  |  |  |  |  |  |
| ***S. hercegovinensis*** | 0.178 |  |  |  |  |  |
| ***S. pretneri*** | 0.159 | 0.209 |  |  |  |  |
| ***S. prasence*** | 0.106 | 0.172 | 0.184 |  |  |  |
| ***S. neglecta*** | 0.158 | 0.166 | 0.084 | 0.157 |  |  |
| ***S. kapelana*** | 0.088 | 0.186 | 0.162 | 0.108 | 0.158 |  |
| ***S. electa sp. nov.*** | 0.211 | 0.157 | 0.206 | 0.183 | 0.193 | 0.205 |
