# Supplementary material for: Revealing European cave shrimp diversity: a new species of Spelaeocaris (Decapoda, Atyidae) named through public participation
Source: Zookeys. 2026 Jan 22;1267:51–76. doi: 10.3897/zookeys.1267.176622 (PMC12856486; doi:10.3897/zookeys.1267.176622)
Supplement: Supplementary material 3 — Morphological data [file zookeys-1267-051_article-176622__-s003.docx]

**Supplementary Table 2.** Comparison of Spelaeocaris electa sp. nov. with its sister species S. hercegovinensis, other species of Spelaeocaris = S. pretneri + S. neglecta + S. kapelana + S. presence and genus Troglocaris = T. anophthalmus, T. planinensis and T. bosnica. Most discriminative features between Spelaeocaris electa sp. nov. and any other species analysed here are presented in bold (note value overlapping in ranges of values; mean ± SD (in brackets) added for a new species and its sister species). Rostral formula presented as in main text.

| **Trait** |  | ***Spelaeocaris electa*  sp. nov.** | ***Spelaeocaris hercegovinensis*** | ***Spelaeocaris* – other species** | ***Troglocaris*** |
| --- | --- | --- | --- | --- | --- |
| References |  | this study | this study, Babić 1922, Stammer 1935, Holthuis 1956, Matjašič 1958, Sket and Zakšek 2009, Jugovic et al. 2010a, 2010b, 2011, unpubl. data | Matjašič 1956a, b, Matjašič 1958, Sket and Zakšek 2009, Jugovic et al. 2010a, 2010b, 2011, unpubl. data | Sket and Zakšek 2009, Jugovic et al. 2010a, 2010b, 2011, 2012, unpubl. data |
| Carapace | spines | supra- and suborbital spines well-developed | supraorbital spines present (**suborbital may be absent)** | with (*T. kapelana*) or **without** *(T. pretneri, T. neglecta, T. prasence)* **supra- and suborbital spines** | supra- and suborbital spines well-developed |
| Rostrum |  | overreaching antennular peduncle (>1.1 times), always with some teeth | in most cases **shorter than antennular peduncle** (but always <1.4 times longer), with or **without teeth** | variable: **shorter than eyestalks and toothless** *(T. neglecta),* **slightly overreaching eyestalks and toothless** *(T. pretneri)* or longer and with teeth (*T. prasence*, *T. kapelana*) | variable: short to very long, always with some teeth |
| Rostrum | rcl ♂♂ | 0.56–0.61 (0.58 ± 0.03); n=2 | **0.17–0.54** (0.35 ± 0.14); n=11 | **0.05–0.57**; n=16 | 0.12–1.07; n=167 |
|  | rcl ♀♀ | 0.49–0.82 (0.70 ± 0.14); n=6 | **0.11–0.57** (0.37 ± 0.14); n=17 | **0.03–0.45**; n=52 | 0.15–1.06; n=327 |
| Rostral formula | ♂♂ | 12+4–6/7–9; n=2–4 | 1–20+1–6/0–6; n=10–11 | 0–14+0–5/0–3; n=15–16 | 0–32+0–14/0–15; n=168–188 |
|  | ♀♀ | 11–17+(3)4–7/5–11; n=8–11 | 0–20+0–11/0–9; n=17 | 0–15+0–7/0–5; n=53 | 0–37+0–17/0–16; n=323–353 |
| Antenna I seg. 1 | apical projection | variable, mostly pointed but short and not jutting out | pointed but short and not jutting out | **blunt and adherent** | sharp, spine-like |
| Maxilliped I | endopodital lobe | narrow and gradually narrowing into a distal flagellum / obliquelly to perpendicularly cut distally, long narrow flagellum | narrow and gradually narrowing into a distal flagellum | very wide, perpendicularly cut or **rounded distally**, flagellum long | obliquelly to perpendicularly cut distally, long narrow flagellum |
| Pereopods | exopodites | I–IV well developed (V: exceptionally developed) | I–IV well developed (V: exceptionally developed) | I–IV well developed (V: missing or rudimentary) | I–IV well developed (V: exceptionally developed, but **always** in ***T. bosnica***) |
|  | robustness | pereopods and their chelae very narow (cf. *S. hercegovinensis*) to narrow (cf. *Troglocaris*) | pereopods and their chelae very narrow | pereopods and their chelae mostly wide and robust | pereopods and their chelae narrow (between *Troglocaridella* and *Spelaeocaris*) |
| Pereopods III-V | ♂ propodus | III–IV differentiated distally | III–IV differentiated distally | III–IV differentiated distally, in ***T. pretneri*** and ***T. neglecta*** **also V** | III–IV differentiated distally |
| Pleopod I | ♂ endopodite | oval and lobate lamina elongated into a finger-like *AI* | oval and lobate lamina elongated into a finger-like *AI* | oval lamina elongated into a finger-like *AI* | **plate-like, wide, *AI* short to distinct, hardly surpassing lamina distally** |
|  | ♂ *PP1AIN* | 12–25 | 13–25 | 0 (juveniles) – 5 (probably subadults) – >50 | **0–5** (exceptionally 7) |
| Pleopod II | ♂ *AM* | *AM* spindle-shaped, numerous short spiniform setae (longest may be longer than *AI* width), *AM* longer and cca 2-times wider than *AI* | *AM* spindle-shaped, numerous **short spiniform setae** (cf. Sket and Zakšek 2009: Fig. 4), *AM* longer and cca 2-times wider than *AI* | *AM* spindle-shaped, with numerous **short spiniform setae**, *AM* longer and cca 2-times wider than *AI* | *AM* shield-like, much wider than *AI*, *AM* >> 3-times longer than *AI*, *AM* with numerous **short spiniform setae** |
| Telson | distal margin | slightly bilobed, concave in the centre | slightly bilobed, concave in the centre | **distinctly convex** | **distinctly convex** |
|  | TET3 ♂♀ | 13–15 (14.00 ± 0.50); n=9 | 10–19 (12.65 ± 1.60); n=26 | 10–16; n=63 | 9–17; n=421 |
| a2s2s1 | ♂♂ | 0.32–0.34 (0.33 ± 0.02); n=3 | **0.33–0.40** (0.37 ± 0.02); n=12 | **0.36–0.55**; n=17 | 0.28–0.41; n=186 |
|  | ♀♀ | 0.30–0.37 (0.33 ± 0.02); n=8 | **0.36–0.43** (0.40 ± 0.02); n=16 | **0.34–0.56**; n=54 | 0.28–0.47; n=348 |
| pr1plmn | ♂♂ | 0.30–0.33 (0.33 ± 0.01); n=3 | **0.26–0.31** (0.28 ± 0.02); n=11 | 0.31–0.47; n=16 | 0.30–0.46; n=181 |
|  | ♀♀ | 0.30–0.33 (0.32 ± 0.01); n=7 | 0.27–0.33 (0.29 ± 0.02); n=16 | 0.31–0.49; n=54 | 0.30–0.47; n=348 |
| s6cl | ♂♂ | 0.52–0.59 (0.55 ± 0.03); n=4 | 0.39–0.56 (0.48 ± 0.05); n=12 | **0.39–0.54**; n=17 | 0.44–0.73; n=189 |
|  | ♀♀ | 0.50–0.57 (0.52 ± 0.02); n=7 | **0.38–0.53** (0.45 ± 0.05); n=17 | **0.36–0.50**; n=54 | 0.34–0.69; n=357 |
| u1te1 | ♂♂ | 1.12–1.27 (1.19 ± 0.07); n=3 | **1.00–1.13** (1.06 ± 0.04); n=9 | 0.99–1.24; n=17 | 0.96–1.38; n=153 |
|  | ♀♀ | 1.08–1.16 (1.14 ± 0.03); n=6 | **0.90–1.08** (1.00 ± 0.05); n=15 | 0.96–1.27; n=45 | 0.90–1.41; n=293 |
| te1s6 | ♂♂ | 0.84–0.95 (0.90 ± 0.01); n=3 | **0.94–1.53** (1.18 ± 0.17); n=10 | **0.93–1.66**; n=17 | 0.75–0.96; n=162 |
|  | ♀♀ | 0.87–0.89 (0.88 ± 0.01); n=5 | **0.98–1.40** (1.18 ± 0.15); n=16 | **0.95–1.51**; n=47 | 0.59–1.20; n=298 |

Abbreviations: n – number of specimens analysed; rcl – ratio: rostral length relative to postorbital carapace length; *PP1AIN* – number of hooks on *appendix interna* of the pleopod I endopodite in males*; AM* – *appendix masculina*, *AI* – *appendix interna*, *TET3* – no. of spiniform setae along distal margin of telson (incl. a pair of distolateral setae; may be used as additional character with other telson traits); a2s2s1 – ratio: width vs. length of scaphocerite; pr1plmn – ratio: pereopod I propodus maximal length vs. pereopod I length; s6cl – ratio: pleonite 6 length vs. carapace length; u1te1 – ratio: uropod length vs. telson length; te1s6 – ratio: telson length vs. pleonite 6 length.

**References**

Babić K (1922) Über die drei Atyiden aus Jugoslavien. Glasnik hrvatskog prirodoslovnog društva 34: 300–306.

Holthuis LB (1956) An enumeration of the Crustacea Decapoda Natantia inhabiting subterranean waters. Vie et Milieu 7: 43-76.

Jugovic J, Prevorčnik S, Sket B (2010a) Development of sexual characters in the cave shrimp genus *Troglocaris* (Crustacea: Decapoda: Atyidae) and their applicability in taxonomy. Zootaxa 21: 1–21. https://doi.org/10.11646/zootaxa.2488.1.1

Jugovic J, Prevorčnik S, Aljančič G, Sket B (2010b) The atyid shrimp (Crustacea: Decapoda: Atyidae) rostrum: Phylogeny versus adaptation, taxonomy versus trophic ecology. Journal of Natural History 44: 2509–2533. https://doi.org/10.1080/00222933.2010.502258

Jugovic J, Prevorčnik S, Blejec A, Sket B (2011) Morphological differentiation in the cave shrimps *Troglocaris* (Crustacea: Decapoda: Atyidae) of the Dinaric karst - a consequence of geographical isolation or adaptation? Journal of Zoological Systematics and Evolutionary Research 49: 185–195. https://doi.org/10.1111/j.1439-0469.2010.00611.x

Matjašič J (1956a) Ein neuer Höhlendecapode aus Jugoslawien. *Zoologischer Anzeiger* 157: 65–68.

Matjašič J (1956b) Eine neue Höhlengarnele aus Herzegowina. *Bulletin Scientifique Yugoslavie* 3: 8.

Matjašič J (1958) O vjetreniških troglocarisih. Biološki vestnik 7: 75-80.

Sket B, Zakšek V (2009) European cave shrimp species (Decapoda: Caridea: Atyidae), redefined after a phylogenetic study; redefinition of some taxa, a new genus and four new *Troglocaris* species. Zoological Journal of the Linnean Society 155: 786–818. https://doi.org/https://doi.org/10.1111/j.1096-3642.2008.00473.x

Stammer HJ (1935) Untersuchungen über die Tierwelt der Karsthöhlengewässer. *Verhandlungen der Internationellen Vereinigung Limnologie* 7: 92–99.
