# Supplementary material for: Revealing European cave shrimp diversity: a new species of Spelaeocaris (Decapoda, Atyidae) named through public participation
Source: Zookeys. 2026 Jan 22;1267:51–76. doi: 10.3897/zookeys.1267.176622 (PMC12856486; doi:10.3897/zookeys.1267.176622)
Supplement: Supplementary material 4 — Detailed comparison of Spelaeocaris electa sp. nov. [file zookeys-1267-051_article-176622__-s004.docx]

**Supplementary Material 4.** Detailed comparison of *Spelaeocaris electa* sp. nov. with its sister species *S. hercegovinensis* and other species of *Spelaeocaris* and *Troglocaris*

Both *Spelaeocaris electa* sp. nov. and *S. hercegovinensis* typically have fewer retinacular hooks on the appendix interna of the male first pleopod (approximately 12–25; Jugovic et al., 2011; present study), compared to other *Spelaeocaris* species (*S. neglecta*, *S. pretneri*, *S. prasence*, *S. kapelana*), which exhibit counts ranging from 5 (in subadult males) to over 50. In contrast, males of *Troglocaris* generally have 0–5 retinacular hooks (exceptionally up to 7) on the appendix interna of pleopod I (Supplementary Table 2). However, it should be noted, that the precise number of hooks – particularly at higher counts – can be difficult to determine with certainty, and that this character is informative only in fully mature males. Spines on appendix masculina of the second male pleopod are dense as in its sister species *S. hercegovinensis*, but at least some of them are quite long, i.e. approximately two times longer than appendix interna’s width of the same pleopod.
